# The Central Role of GSNOR: Decoding Nitric Oxide Signaling for Crop Stress Tolerance

**DOI:** 10.3390/ijms262311486

**Published:** 2025-11-27

**Authors:** Ashim Kumar Das, Da-Sol Lee, Geum-Jin Lee, Ye-Song Kim, Sajeel Hussain, Moon-Sub Lee, Byung-Wook Yun, Bong-Gyu Mun

**Affiliations:** 1 Department of Applied Biosciences, College of Agriculture and Life Sciences, Kyungpook National University, Daegu 41566, Republic of Korea; 2 Department of Crop Science, Chungbuk National University, Cheongju 28644, Republic of Korea; 3 Department of Environmental and Biological Chemistry, Chungbuk National University, Cheongju 28644, Republic of Korea

**Keywords:** nitric oxide, abiotic stress, biotic stress, GSNOR, nanoparticles, signaling mechanisms

## Abstract

S-nitrosoglutathione (GSNO) reductase (GSNOR) is a major and conserved enzyme in prokaryotes and eukaryotes. It reduces a stable nitric oxide (NO) reservoir, GSNO, to balance the organisms’ redox status through S-nitrosylation. Over the last few decades, much of our understanding of GSNOR’s roles in plant biology has been updated. Here, therefore, we review the current knowledge of GSNOR in plant physiology and signaling under abiotic and biotic stresses. We observe that the role of GSNOR in plant abiotic stress is widely studied in both model and crop plants, whereas studies on its role in biotic stress have mainly focused on model plants. Under abiotic stresses, GSNOR plays a pleiotropic role in terms of plant tolerance and sensitivity. The presence or absence of GSNOR activity modulates the endogenous NO pool that balances plant reactive nitrogen species (RNS) and reactive oxygen species (ROS) under stress conditions. Moreover, GSNOR regulates hormonal levels, like ethylene, abscisic acid (ABA), jasmonic acid (JA), and salicylic acid (SA), in response to abiotic and biotic stress conditions. Although GSNOR is important in plant physiology, its regulation of the redox switch is directly influenced by the extent of S-nitrosylation, where S-nitrosylated proteins generally enhance plant tolerance to abiotic stress but simultaneously suppress plant immunity. We further highlight a new perspective on NO-based nanotechnology in agriculture, focusing on GSNO encapsulated in nanocarriers. This technology improves NO stability and opens new avenues by allowing an evaluation of GSNOR’s role for sustainable crop production. Intriguingly, we discuss knowledge gaps, which are crucial to understanding the role of GSNOR in plant stress tolerance. Overall, this review accumulates a comprehensive understanding of the GSNOR enzyme in crop biology, which could aid in harnessing its function to address the impacts of climate change.

## 1. Introduction

### 1.1. Nitric Oxide

**Figure 1:**
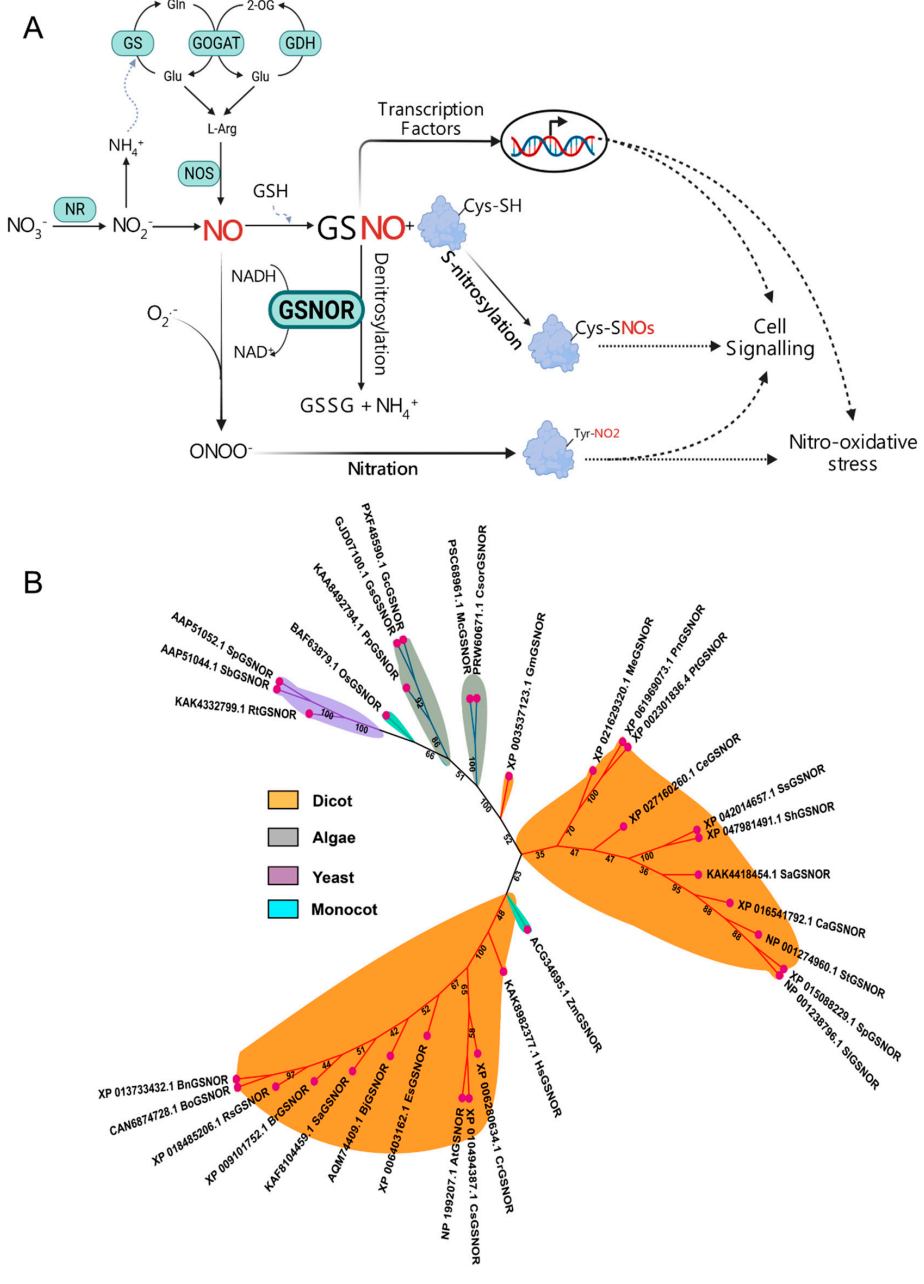
(**A**) Schematic of nitric oxide (NO) signaling and associated pathways. NO is produced via different pathways in mammals and plants. In mammals, it is generated through an oxidative pathway by nitric oxide synthase (NOS), while in plants, a reductive pathway involving nitrate reductase (NR) is a major source. NO reacts with glutathione (GSH) in an O_2_-dependent manner to form S-nitrosoglutathione (GSNO). GSNO functions as a stable NO reservoir and carrier, transferring its NO moiety to the sulfhydryl (-SH) group of a cysteine residue on target proteins. This process is called S-nitrosylation, a unique redox-based post-translational modification that forms S-nitrosothiols (SNOs). In contrast, the conserved enzyme GSNO reductase (GSNOR) facilitates de-nitrosylation by reducing GSNO to glutathione disulfide (GSSG) and ammonium (NH_4_^+^) using an electron donor from NADH. The balance between S-nitrosylation and de-nitrosylation is crucial for regulating diverse biological processes. Figure was created with the software BioRender (https://www.biorender.com/). (**B**) An unrooted phylogenetic tree was prepared using the neighbor-joining method with a bootstrap value of 1000 in the Mega tool and iTOL (https://itol.embl.de/, accessed on 15 September 2025). The protein sequences were downloaded from the NCBI database (https://www.ncbi.nlm.nih.gov/, accessed on 10 September 2025) and used to analyze the evolutionary relationships among the GSNOR orthologs.

Nitric oxide (NO) is a short-lived, diatomic gas molecule with a redox-active nature that is produced by one cell, and it penetrates membranes to regulate the functions of other cells [1,2]. Initially, it gained prominence as a key signaling molecule for muscle relaxation, identified by endothelium-derived relaxing factor (EDRF) [3,4,5]. Even before that, Priestley [6] reported that NO consists of a single oxygen and nitrogen. Meanwhile, Klepper and his team observed the possible NO evolution from soybean and winged bean plants in vivo through nitrate reductase (NR) assay [7,8,9]. While the primary source of mammalian NO is nitric oxide synthase (NOS), which converts L-arginine to NO and citrulline [10], a homologous gene has not been found in plants [11]. Instead, plant NO production is mediated by the nitrate reduction pathway, which is catalyzed by NR, which reduces nitrate to nitrite and then to NO (Figure 1A) [12,13,14]. Few studies still hypothesize that plants possess NOS-like activity for NO production [15,16]. Hence, researchers are still in the race for identifying the additional occurrence and evolution of NO production in plants [17]. Although its synthesis differs between mammals and plants, NO functions as a paramount signaling molecule in a wide range of biological processes. However, we have little knowledge of how precisely NO signaling regulates crop yield traits to advance agricultural production. Ongoing research continues to reveal additional regulators and mechanisms, expanding our understanding of the NO-mediated complex network. In this review, we highlight how S-nitrosoglutathione (GSNO) reductase (GSNOR) regulates NO response in plants.

### 1.2. An Introduction to the GSNOR Enzyme and Its Role in GSNO Turnover

NO reacts with glutathione (GSH) through an O_2_-dependent reaction to form GSNO, which functions as a stable reservoir and carrier of NO (Figure 1A) [18,19]. Unlike the short-lived NO, GSNO is likely mobile within the phloem, enabling it to travel long distances to redox signaling [20,21]. GSNO transfers its NO moiety to the sulfhydryl (-SH) group of a cystine residue on target proteins to form low-molecular-mass S-nitrosothiol (SNO) (Figure 1A). This process, called protein S-nitrosylation, is a unique redox-based post-translational modification (PTM) [22,23,24,25]. SNO can further transfer the NO moiety to the -SH of other cystine proteins to form high-molecular-mass SNOs, known as thiol-to-thiol SNO formation (S-transnitrosylation) [25,26]. The stability of GSNO necessitates a mechanism for its controlled turnover. This function is critically managed by the enzyme GSNOR, which acts as the key governor of S-nitrosylation. Over two decades ago, GSNO turnover by GSNOR was first reported in purified *Escherichia coli*, which is similarly thought to be a reducing agent of GSNO in yeast and mice [27]. GSNOR uses the electron donor NADH as a cofactor to reduce GSNO into glutathione disulfide (GSSG) and ammonium (NH_4_^+^), which ultimately reduces the cellular levels of GSNO and SNO (Figure 1A) [28]. An ortholog of this single protein (AtGSNOR1; AT5G43940; 379 residues) was also identified in *Arabidopsis thaliana*, which was originally a class III alcohol dehydrogenase (ADH3, EC 1.1.1.1)/glutathione-dependent formaldehyde dehydrogenase (FALDH, EC 1.2.1.1) [29,30]. Even a decade before, Koivusalo et al. [31] found that ADH3 and FALDH are identical enzymes. Loss-of-function mutation in AtGSNOR1 (*Atgsnor1-3*) results in higher cellular levels of both GSNO and total SNO. In contrast, gain-of-function (*Atgsnor1-1*) exhibited elevated GSNOR activity with a marked reduction in GSNO and SNO [30]. Thus, GSNOR not only turns over cellular GSNO, but, by doing so, also depletes the total SNO pool, which then limits the extent of protein S-nitrosylation (Figure 1A).

To understand the evolutionary map of GSNOR across dicots, monocots, algae, and yeast, we performed a phylogenetic tree analysis. The tree showed that GSNOR maintains an evolutionary relationship across these species (Figure 1B). Additionally, we employed a multiple sequence alignment by Clustal W (SnapGene 8.1.0) that showed a high level of sequence conservation (>95%) across all selected species (Table S1; Figure S1). Notably, a total of nine highly conserved cystine regions were observed among monocots, dicots, and algae, including Cys10, Cys47, Cys99, Cys102, Cys105, Cys113, Cys178, and Cys272 (Figure S1). These results suggest that GSNOR is a conserved protein in plant species, potentially a crucial regulator of S-nitrosylation and de-nitrosylation. The regulatory mechanism of S-nitrosylation is a rapidly updating field of research, as its ubiquitous involvement in biological processes becomes increasingly clear. This review, therefore, racks up a series of studies that have investigated S-nitrosylated proteins in plants under various growth and stress conditions (Table 1). These investigations commonly employed a biotic switch assay coupled with a liquid chromatography–tandem mass spectrometry (LC-MS/MS) analysis system [32,33]. In the following sections, we discuss how the S-nitrosylation of these proteins affects the function of GSNOR in plants or vice versa. Moreover, we noticed that multiple previous discussions were solely focused on S-nitrosylation in diverse proteins [25,34,35] or inadequately addressed the multifaceted role of GSNOR in plants [14,36,37,38]. Therefore, this review discusses the role of GSNOR in plant stress responses by integrating up-to-date findings and knowledge gaps from studies that used genetic and exogenous approaches to modify cellular GSNOR activity and GSNO levels.

**Table 1:** List of S-nitrosylation sites and detection methods in *A. thaliana* and crops under abiotic and biotic stress.

Species	S-Nitrosylation Sites	Target Protein	Upstream Signal	Detection Method	Reference
*Arabidopsis thaliana*	Cys-80	BIK1	P/MAMPs	Biotin switch assay	[39]
*Arabidopsis thaliana*	Cys-425, Cys-607	COP1	Light	Biotin switch assay, nano-LC-MS/MS	[40]
*Arabidopsis thaliana*	-	QSOX1	Heat stress	Biotin switch assay	[41]
*Arabidopsis thaliana*	Cys-337	ERO1	ER stress	TMT labeling, LC-MS/MS	[42]
*Arabidopsis thaliana*	Cys-137	HDA19	Oxidative stress	Biotin switch assay, TMT labeling, LC-MS/MS	[43]
*Arabidopsis thaliana*	Cys-374	RGA	Salt stress	Biotin switch assay, LC-MS/MS	[44]
*Arabidopsis thaliana*	Cys-164	HFR1	High temperature	Biotin switch assay	[45]
*Arabidopsis thaliana*	-	AtNRAMP3, AtNRAMP4, AtPIC1	Iron deficiency	GPS-SNO 1.0 software (in silico and protein stability assay)	[46]
*Arabidopsis thaliana*	Cys-10	GSNOR1	Hypoxia	Biotin switch assay, DAN Assay, LC-MS/MS	[47]
*Arabidopsis thaliana*	Cys-137	SnRK 2.6	Drought	Biotin switch assay, LC-MS/MS	[48]
*Arabidopsis thaliana*	Cys-32	APX1	Oxidative stress	Biotin switch assay, DAN assay, LC-MS/MS	[49]
*Arabidopsis thaliana*	Cys-890	RBOHD	Pathogen	Biotin switch assay, LC-MS	[50]
*Arabidopsis thaliana*	Cys-28	AtSABP3	Pathogen infection	Biotin switch assay, LC-MS/MS	[51]
*Arabidopsis thaliana*	Cys-156	NPR1	Pathogen infection	Biotin switch assay	[52]
Tomato *(Solanum lycopersicum)*	Cys-316, Cys-258, Cys-316	SlGABA-TP1, SlGABA-TP2, SlGABA-TP3	Saline-alkaline stress	Biotin switch assay	[53]
Tomato *(Solanum lycopersicum)*	Cys-5	SlP5CR	Drought and salt stress	Biotin switch assay	[54]
Tomato *(Solanum lycopersicum)*	Cys-54	SlTrxh	Nitrate stress	Biotin switch assay, LC-MS/MS	[55]
Tomato *(Solanum lycopersicum)*	Cys-172	ACOh4	Salt stress	Biotin switch assay, LC-MS/MS	[56]
Mini Chinese Cabbage (*Brassica rapa* ssp. *pekinensis*)	-	BrGSNOR	Low temperature stress	Biotin switch assay	[57]
Peach (*Prunus persica* (L.) Batsch)	Cys-85	*-*	Pathogen infection	Iodo-TMT labeling, LC-MS/MS	[58]

Full abbreviations from the table can be found in the main text or Abbreviation section.

## 2. Changes in GSNOR Activity Affect Abiotic Stress Tolerance

Given the multifaceted role of GSNOR in plant growth, it is also vital for balancing endogenous NO levels and regulating signaling in the plant response to abiotic stress [36,59,60]. The multitude of NO responses in plants under abiotic stress poses a major challenge to understanding their precise signaling mechanisms. Additionally, the function of GSNOR in enabling plant stress adaptation is being widely studied, focusing on responses to potential agricultural devastation caused by climate-change-driven catastrophes. In this section, we provide an update on core GSNOR-mediated NO signaling mechanisms connected with signaling transductions under various abiotic stresses.

### 2.1. High- and Low-Temperature Stress Tolerance

**Figure 2:**
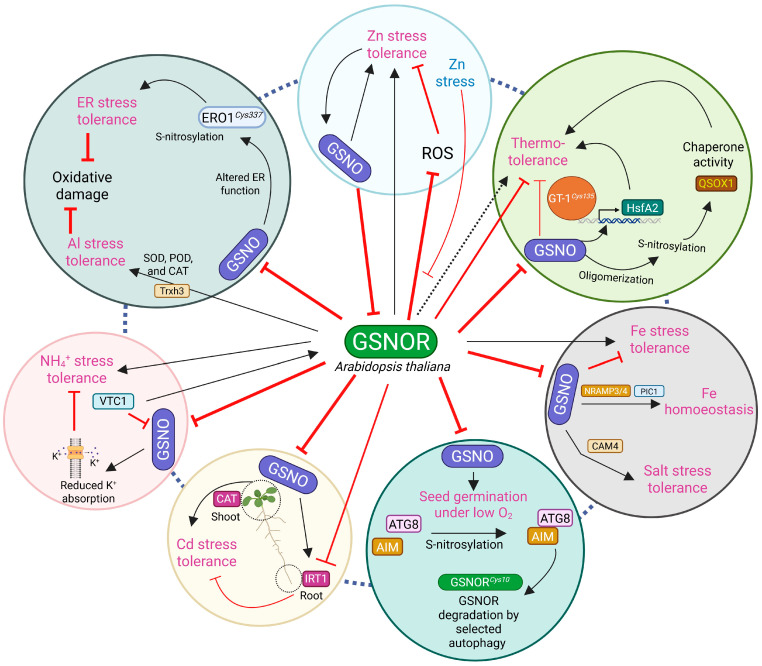
This figure illustrates the role of S-nitrosoglutathione (GSNO) reductase (GSNOR) in mediating tolerance in *A. thaliana* across a wide range of abiotic stresses, including thermo-tolerance, iron homeostasis and tolerance, salt, drought, hypoxia, and cadmium (Cd) and aluminum stress, as well as tolerance to metal and nutritional stress (NH_4_^+^ and Zn), and endoplasmic reticulum (ER) stress. A pleiotropic effect of GSNOR has been noticed, where plant tolerance with higher or lower levels of GSNOR activity varies depending on stress conditions and their signaling mechanisms. Black solid or dash arrows indicate direct or indirect activation where red blunt shows inhibition. All the abbreviations are available in the main text.

**Figure 3:**
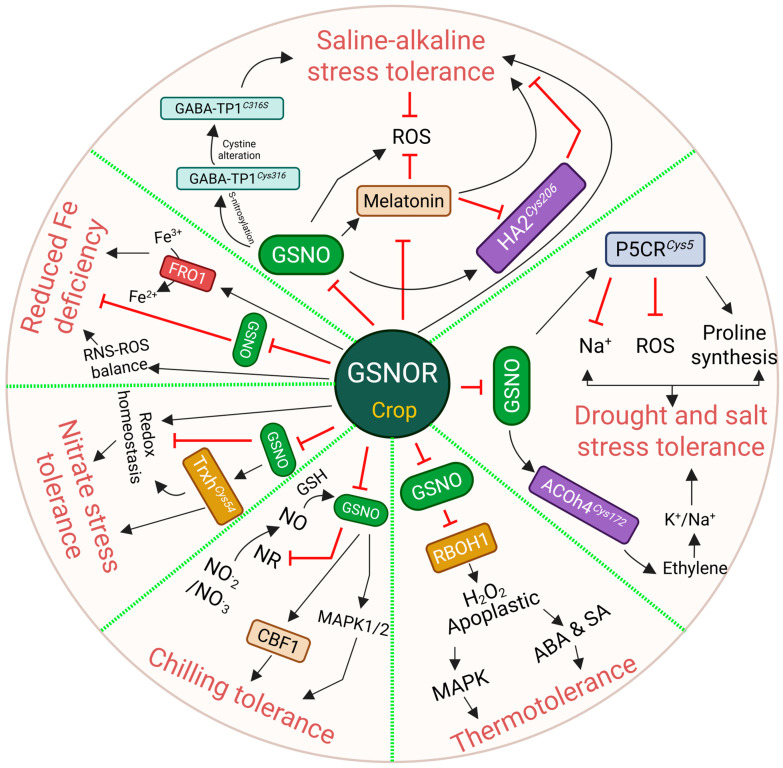
This figure illustrates the role of S-nitrosoglutathione (GSNO) reductase (GSNOR) in mediating tolerance in crops across a wide range of abiotic stresses, including temperature tolerance, iron homeostasis, and salt and drought stress, as well as tolerance to nitrate stress and saline–alkaline stress. A pleiotropic effect of GSNOR has been noticed, where crop tolerance with higher or lower levels of GSNOR activity varies depending on stress conditions and their signaling mechanisms. GSNOR modulates the reactive oxygen species (ROS)-reactive nitrogen species (RNS) balance and redox switches through S-nitrosylation under abiotic stresses. Black solid arrows indicate direct activation where red blunt shows inhibition. All the abbreviations are available in the main text.

This review explored differences in the thermotolerance of endogenous NO and GSNO levels in *A. thaliana*, which were then related to GSNOR activity. NO-deficient Arabidopsis mutants, *noa1* and *nia1nia2*, showed an enhanced heat acclimation response [61]. Conversely, the GSNOR null mutant, *hot5-2/4*, which accumulates excessive GSNO, displayed high heat sensitivity, suggesting that elevated RNS through nitrosative stress blunts heat acclimation (Figure 2; Table 2) [62]. A consistent result was observed in tomato plants with suppressed *SlGSNOR* activity. This compromised heat acclimation was linked to several molecular changes: reduced levels of ABA and salicylic acid (SA) and decreased activation of mitogen-activated protein kinase (MAPK), heat shock protein 90 (HSP90), and respiratory burst oxidase homolog 1 (RBOH1) (Figure 3; Table 3) [63]. Consequently, the suppressed *SlGSNOR* lines failed to produce apoplastic H_2_O_2_. H_2_O_2_ plays an important role in the early activation of heat shock proteins [64], and potentiating GSNOR is essential for maintaining redox balance and achieving thermotolerance. Intriguingly, although the broad loss of GSNOR activity compromised thermotolerance, the reduced GSNOR activity of plant quiescin sulfhydryl oxidase homolog (QSOX1) led to enhanced heat tolerance (Figure 2) [41,65]. The targeted S-nitrosylation of QSOX1 switched from an oxidoreductase to a molecular chaperone, leading to heat resistance (Table 1). Similarly, NO burst in a member of the trihelix transcription factor, GT-1, was S-nitrosylated and bound in the promoter of HsfA2 to activate heat-responsive expression (Figure 2; Table 1) [66]. While *hot5-2/4* or silenced *SlGSNOR* plants lost heat tolerance, elevated GSNO or S-nitrosylation of other proteins may not inherently be negative (Table 1). Supportively, it is plausible that exogenous NO application increases heat tolerance [67].

Similar to heat tolerance, exogenous NO application has recently gained research focus in cold stress acclimation [68,69], as low temperature causes detrimental effects on crop growth and development. The NR null mutant, *nia1nia2*, failed to undergo cold acclimation, which was restored by the supplementation of SNP [70]. Conversely, Costa-Broseta et al. [71] reported that Arabidopsis *nia1nia2noa1-2* were insensitive to cold stress. These discrepancies need to be revisited using both double and triple mutants simultaneously to check whether NOA1-dependent NO regulation has any effect on cold acclimation or whether the observed effects are related to the stress duration, severity, and experimental conditions. However, no studies have been performed using GSNOR null mutants, like *gsnor1-3/hot5-2/4*, to evaluate cold tolerance. Therefore, null mutants of NR and NOA failed to mimic the role of GSNOR-mediated NO elimination for cold tolerance in *A. thaliana*. In response to crops, silencing of tomato *GSNOR* resulted in MAPK1/2 and C-repeat binding factor (CBF1) activation, which helped in cold stress acclimation (Figure 3; Table 3) [72]. Moreover, brassinosteroid-dependent S-nitrosylation of monodehydroascorbate reductase (MDHAR) also confers low-temperature tolerance by improving ROS detoxification (Table 1; Table 3) [73]. These findings led us to assume that the loss of GSNOR activity or elevated NO may improve freezing resilience, a notion supported by many studies on exogenous NO application. Moreover, we also hypothesize that the loss-of-GSNOR mutation in *gsnor1-3*/*hot5-2/4* likely exhibits cold tolerance.

### 2.2. Iron Stress Tolerance and Homeostasis

NO has a high affinity for iron (Fe) that can react with ferric (Fe^3+^) and/or ferrous (Fe^2+^) forms of Fe [74]. Parallelly, NO and Fe can form an iron–nitrosyl complex that is essential for Fe homeostasis [75]. Excessive Fe is toxic to biological processes, as it reacts with H_2_O_2_ to generate highly destructive hydroxyl radicals (OH^•^) [76]. NO-mediated oxidative burst is also a reason for nitrosative stress and exerts severe cellular damage [36]. Examining the previous studies, we observed that the role of GSNOR activity or GSNO levels is highly varied in regulating Fe toxicity and homeostasis. Li et al. [77] reported that GSNOR is essential for plant tolerance to high Fe, as evidenced by *hot5-2/4* or silenced *GSNOR* activity of *Lotus japonicus* and rice (*Oryza sativa* L.) plants, which were highly sensitive to Fe toxicity (Figure 2 and Figure 3; Table 2 and Table 3). In contrast, overexpression of tomato *GSNOR* plants mitigated Fe deficiency by upregulating the transcript levels of Fe absorption and transportation, including ferric-reductase oxidase 1 (FRO1) and oligopeptide transporter (OPT), respectively [78]. Moreover, GSNOR expression and Fe acquisition genes were upregulated under Fe-deficient conditions in *A. thaliana* [79]. These results suggest that the presence of GSNOR activity alleviates excessive RNS and ROS, thereby reducing cellular damage and improving Fe homeostasis to better tackle Fe toxicity (Figure 2 and Figure 3; Table 2).

On the other hand, *gsnor1-3* plants exhibited a higher relative expression of vacuolar Fe (*NRAMP3* and *NRAMP4*) and chloroplast Fe importer (*PIC1*) under Fe-deficient conditions (Figure 2; Table 2) [46]. This result contrasts with the positive regulation of GSNOR activity, but Shee et al. [46] discussed that elevated GSNO in *gsnor1-3* conferred Fe transportation, mainly dependent on reduced glutathione (GSH). While GSNO is formed from cellular NO and GSH and participates in diverse signaling mechanisms, mutants lacking these precursor molecules—the NO-deficient *noa1* and GSH-deficient *cad2-1*/*pad2-1*—both exhibited hypersensitivity to Fe deficiency. Hence, both GSH and NO levels are likely crucial for Fe homeostasis in plants. However, it is unclear why the loss of GSNOR simultaneously makes a plant hypersensitive to high Fe but upregulates Fe transport genes during Fe deficiency. Answering with a unifying role of GSNOR under Fe levels will require simultaneous monitoring of different species and conditions.

**Table 2:** GSNOR-mediated regulation of abiotic stress tolerance in *A. thaliana*.

Mode of Genetic Modification	Stress Conditions	GSNOR Activity/Expression	NO/SNO Levels	Crosstalk with Other Proteins	Stress Effects	ROS: Antioxidant	Crosstalk with Hormones	Reference
*hot1*	Heat	–	–	QSOX1	Tolerance	–	–	[41]
*gsnor1-3*	Cd	–	–	–	Tolerance	↓:↑	–	[80]
*GSNOR*					Sensitive	↑:↓		
*hot5-4*	ER	–	–	ERO1	Tolerance	–	–	[42]
*gsnor1*	Oxidative	–	–/Increased	HDA19	–	–	–	[43]
*hot5-2*	Heat	–	Increased/–	GT-1	Tolerance	–	–	[66]
*hot5-2*	NH_4_^+^	–	Increased/–	–	Sensitive	–	–	[81]
*gsnor1-3*	Oxidative	–	–	–	Sensitive	–	–	[82]
*gsnor1-3*	Light intensity	–	Increased/Increased	HDA6	–	–	–	[83]
*gsnor1-3*	Oxidative	Inhibited	–/Increased	ICS1	–	–	SA	[84]
*gsnor1-3*	Oxidative	–	–	ROG1	Tolerance	–	–	[85]
*gsnor1-3*	Zn	–	–	APX1	Tolerance	↑:↓	–	[86]
*35S:FLAG-GSNOR1*		Inhibited	Decreased/Increased		Sensitive	↑:↓		
*gsnor1-3*	Cd	–	–	IRT1 and APX	Sensitive	–	–	[87]
*GSNOR*		Induced			Tolerance			
*gsnor1-3*	Fe	–	Increased/–	–	Sensitive	–	–	[77]
*gsnor1-3*	Hypoxia	–	–	ATG8	Sensitive	–	–	[47]
*gsnor*	Salt	Inhibited	Increased/–	CaM	Tolerance	–	–	[88]
*GSNOR*		Induced	Decreased/–		Sensitive			
*gsnor1-3*	Oxidative	–	–	APX1	Tolerance	–:↑	–	[49]
*gsnor1*	Nitrate	Inhibited	–	–	Sensitive	–	–	[89]
*35S:FLAG-GSNOR1*		Induced			Tolerance			
*hot5*	Heat	–	–	–	Sensitive	–	–	[62]

Full abbreviations from the table can be found in the main text or Abbreviation section. ↑, increased; ↓, decreased.

### 2.3. Salt, Drought, and Metal Stress Tolerance

NO has a paramount role in regulating plant salt, drought, and heavy metal stress tolerance [90]. In this section, we discuss how GSNOR modulates plant physiology and signaling under these stress conditions. Zhou et al. [88] reported that GSNOR negatively regulates salt tolerance, as the Arabidopsis *gsnor* mutant showed enhanced survivability to 100 mM NaCl. Moreover, the increased GSNOR activity in Ca^2+^ sensor protein knockout mutants, *cam1* and *cam4*, effectively abridged NO levels, resulting in salt sensitivity. In contrast, *cam4gsnor* plants exhibited higher survivability under NaCl stress, pinpointing that NO acts as a downstream regulator of Ca^2+^ signaling to modulate salt tolerance (Figure 2; Table 2) [88]. Furthermore, *GSNOR RNA interference (RNAi)* tomato plants increased ethylene and NO accumulation under salt stress, while *GSNOR-overexpressed (OE)* plants showed a compromise of both [56]. Elevated ethylene and NO, together, fight against salt toxicity by upregulating 1-aminocyclopropane-1-carboxylate (ACC) synthase (ACS) and ACC oxidase (ACO) activity and their relative mRNA expression. Later, it was shown that S-nitrosylation of ACOh4*^Cys172^* maintained K^+^/Na^+^ homeostasis to promote salt stress tolerance (Figure 3; Table 1 and Table 3). Recently, the same research team reported that the S-nitrosylation of Δ^1^-pyrroline-5-carboxylate reductase (SlP5CR*^Cys5^*) enhanced salt and drought tolerance by boosting proline synthesis while limiting the ROS and Na^+^ accumulation [54]. Similar to SlP5CR*^Cys5^*, *SlGSNOR RNAi* lines exhibited higher proline accumulation. Although proline is known for its role in the mitigation of abiotic stress tolerance [91], its intersection with NO remained elusive. Thus, this study first elucidated the crosstalk of proline and NO responses. Overall, these findings suggest that GSNOR plays a negative role in Ca^2+^, ethylene, and proline accumulation to protect plants from salt stress (Figure 3; Table 1 and Table 3).

During drought stress, ABA signaling is crucial for regulating stomatal movement, which maintains plant transpiration rate and drought acclimation. However, NO acts as a negative regulator of ABA signaling by S-nitrosylating the protein SnRK2.6*^Cys137^*, thereby limiting its activation (Table 1) [48]. Consistent with this, the *GSNOR* knockout mutant, *gsnor1-3*, over-accumulates NO in guard cells and consequently impairs stomatal closing (Table 2). This suggests that NO-mediated inhibition of ABA signaling is a critical factor limiting drought tolerance, making the GSNOR gene a key component in plant drought resilience. Despite the contrasting roles of GSNOR in plant salt and drought stress tolerance, GSNOR showed tissue-specific heavy metal tolerance in *A. thaliana*. Previously, it was reported that GSNOR limits Cd^2+^ accumulation in roots by restricting *iron-regulated transporter 1* (*IRT1*), which is a Cd transporter (Figure 2; Table 2) [87]. A few years later, the same group mentioned that GSNOR disfavors Cd tolerance, as *gsnor1-3* plants acquired less ROS with higher CAT activity in the shoots (Figure 2; Table 2) [80]. Similarly, *gsnor1-3* also increased APX activity under oxidative stress, indicating its effective ROS detoxification mechanism (Table 2) [49]. Nevertheless, the conflicting data present a paradox: how can GSNOR limit Cd^2+^ accumulation in the roots while its absence still results in enhanced metal-dependent ROS production in the leaves? Therefore, this limited knowledge needs to be re-investigated.

### 2.4. Nutrient Stress Tolerance

Nutrients are available in the soil and are essential for plant growth and development. However, inadequate or excessive fertilization in agricultural lands often causes nutrient pollution or toxicity that restricts crop production. Hence, it is crucial to understand the plant physiology and mechanisms occurring under nutrient stress conditions. Nitrogen assimilation is one of the core metabolic processes in plant growth, which is carried out through the nitrate reduction pathway (Figure 1A). Nitrogen assimilation is largely connected with the formation of NO, which regulates plant development and stress responses. Frungillo et al. [89] reported that elevated NO and GSNO differentially modulate nitrogen assimilation by inhibiting nitrate uptake and reduction. Specifically, nitrate-derived NO accumulation represses GSNOR activity, suggesting that NO is the key to the adjustment of nitrogen assimilation. Additionally, S-nitrosylation of tomato SlTrxh*^Cys54^* alleviates ROS accumulation under nitrate stress [55], indicating a similar NO-induced GSNOR inhibition that likely regulates nitrogen pollution due to over-fertilization. In stark contrast, overexpression of spinach (*Spinacia oleracea* L.), *SoGSNOR*, exhibited improved ROS and RNS balance—enhancing the plants’ germination rate under nitrate stress (Figure 2; Table 3) [92]. Therefore, it would be more informative to be able to justify the role of GSNOR transgenic lines of either tomato or other crops under nitrate stress.

Although NH_4_^+^ is less demanded by plants than nitrate, its moderate presence in the soil can cause toxicity directly to plant roots [93]. Plant hypersensitivity to NH_4_^+^ was observed due to disturbed NO signaling in the knockout of the *vitamin C1* (*VTC1*) mutant [94]. A recent study explored GSNOR’s roles in Arabidopsis and rice under NH_4_^+^ stress [81]. The study exhibited that *hot5-2/gsnor* and *Osgsnor-1/2* plants showed hypersensitivity to NH_4_^+^ stress, similar to *vtc1-1* plants. This sensitivity reduced the root length and K^+^ adsorption; however, a double mutant of *35S-GSNOR/vtc1-1* restored the root length, suggesting a GSNOR-dependent positive role of plants’ NH_4_^+^ stress tolerance (Figure 2; Table 2). Plants exposed not only to nitrate and NH_4_^+^ stress, but also to excess Zn-rich soil experience altered normal growth and development, including inward rolled leaf edges, chlorotic leaves, and retarded and brownish root systems [95]. In addition, excess Zn accumulation resulted in overaccumulation of RNS, including NO [96], which was then responsible for GSNOR inactivation and reduced Zn tolerance [86]. This inactivation directly accumulated higher H_2_O_2_ by reducing glutathione and APX activities. Contrarily, *35S:FLAG-GSNOR1* plants compensated for suboptimal Zn toxicity by reducing H_2_O_2_ formation (Figure 2; Table 2) [97]. Taken together, these findings indicate that the function of GSNOR in Zn toxicity is conditional, varying with the specific Zn levels. This difference underscores the necessity of considering soil characteristics when defining GSNOR’s exact role under Zn stress.

In this review, we examine the pleiotropic role of GSNOR in sensing nutrient toxicity. It is also crucial to understand how GSNOR activity adapts to nutrient deficiency or how plant roots with altered GSNOR activity modulate microbial activity in the rhizosphere in relation to nutrient availability, uptake, and translocation. Moreover, elucidating the role of GSNOR in a phosphorus-deficient medium will be interesting, as phosphorus is the most limiting nutrient in soil owing to its insolubility.

### 2.5. Saline–Alkaline Stress Tolerance

Saline–alkaline stress is defined by the presence of alkaline salts, primarily NaHCO_3_ (sodium bicarbonate) and Na_2_CO_3_ (sodium carbonate), in the soil. Globally, saline–alkali soils cover 954 million hectares—impacting global agriculture—as shown by Liu et al. [53]. Plants manage saline–alkaline stress through organic acid metabolism that helps in detoxifying Na^+^, regulating rhizosphere pH, maintaining osmotic balance, and managing ROS accumulation [98,99]. Over the last decades, the role of NO signaling in saline–alkaline stress tolerance has been updated through the redox switch of the GSNOR enzyme. Gong et al. [100] and Wei et al. [101] reported that *GSNOR*-overexpressed tomato lines were tolerant, while *GSNOR*-suppressed plants were hypersensitive (Figure 2; Table 3). Although the tolerant lines were found to coordinate ROS and RNS balance, the sensitive *GSNOR*-suppressed lines presented a paradox, as they displayed a reduced Na^+^/K^+^ ratio and increased expression of salt marker genes [100]. However, the same group recently revealed new findings, where the *GSNOR RNAi* line accumulated higher Na^+^, as well as saline–alkaline hypersensitivity (Figure 2; Table 3) [101]. This conflicting data may be a reason for the differential genetic modification. Despite the higher saline–alkaline sensitivity in *GSNOR RNAi* lines, these were salt-tolerant, as we discussed in the previous sections. While elevated GSNO levels in *GSNOR RNAi* lines potentially drive global S-nitrosylation, how this redox switch manages Na^+^ toxicity but fails to compensate for high pH stress was recently elucidated with malate exudation mechanisms.

Pyruvate-dependent GABA transaminase 1 (SlGABA-TP1) was found to be S-nitrosylated at Cys316/258/316, and increasing γ-aminobutyric acid (GABA) reduces saline–alkaline tolerance through the reduction in malate exudation (Figure 2; Table 3) [53]. Intriguingly, when S-nitrosylation is blocked by mutating the target cysteine residues to serine, the resulting change in NO levels supports malate movement in both roots and fruits, ultimately increasing overall stress tolerance. Elevated root GABA levels in *GSNOR RNAi* were also observed, suggesting that excess GABA reduces malate exudation in this line, which is essential for saline–alkaline stress adaptation (Figure 2; Table 3). Similar to the PTM of SlGABA-TP1, S-nitrosylated plasma membrane-localized H^+^-ATPase 2 (HA2*^Cys206^*) limits its interaction with 14-3-3 protein 1 (TFT1)—resulting in impaired HA activity, H^+^ efflux, and thereby increased saline alkaline sensitivity (Figure 2; Table 3) [101]. However, this sensitivity is reduced with the help of melatonin, an important phytohormone. *GSNOR RNAi/COMT OE* lines exhibited saline–alkaline tolerance. COMT (caffeic acid O-methyltransferase) is one of the catalyzing enzymes that facilitates the melatonin biosynthesis from tryptophan [102]. In contrast, *GSNOR RNAi* lines were hypersensitive but still accumulated higher melatonin levels, whereas *GSNOR OE* failed to do so [101]. Thus, this suggests that the detrimental effects of GSNO/NO dysregulation in the mutant likely overrode the benefits of the elevated melatonin.

### 2.6. Other Stress Responses

The role of GSNOR has also been studied in plants under hypoxia, aluminum, and endoplasmic reticulum (ER) stress. However, we know little about their crosstalk with GSNOR, where these stress conditions hold importance by their own mode of action on plant physiology and signaling transduction. Hypoxic conditions in plants represent a major concern due to climate change, often resulting from heavy flash flooding. Despite NO’s roles in the regulation of plant metabolism under hypoxic or anoxic conditions [103], before the study published by Zhan et al. [47], the function of GSNOR in mediating plant response to hypoxic stress was unknown. They reported that S-nitrosylated GSNOR*^Cys10^* activates the selective autophagy of GSNOR by exposing its autophagy-related protein 8 (ATG8)-interacting motif (AIM) (Figure 2; Table 2). This autophagy was found to be relevant to hypoxia responses. In particular, under 3% O_2_ levels, despite the marked upregulation of two hypoxia marker genes, *alcohol dehydrogenase 1* (*ADH1*) and *pyruvate decarboxylase 1* (*PDC1*), in *gsnor1-3*, it showed hypersensitivity during germination. This pleiotropic effect was likely caused by a combination of factors, including inefficient O_2_ utilization, a mechanism that is independent of GSNOR, and ABI5 downregulation. Conversely, the NO overproducer *nox1* mutant exhibited enhanced germination and higher *PDC1* expression. This finding implies that NO itself promotes seed germination under low-oxygen conditions, a mechanism distinct from the GSNO-driven effects seen in *gsnor1-3*. Despite the previously established negative role of NO under low-O_2_ conditions, the observation that NO promotes germination seems contradictory and is difficult to reconcile—a challenge that Zhan et al. [47] addressed by suggesting that this alteration may focus primarily on ABA signaling.

In acidic soils, aluminum is solubilized and available for plants in Al^3+^ and Al(OH)^2+^ forms, inhibiting plant growth and development. Peanut (*Arachis hypogaea* L.) GSNOR1 transcription and protein expression were enhanced under aluminum stress. An introgressed *AhGSNOR1*-overexpressing transgenic tobacco plant decreased aluminum-induced NO and SNO accumulation, while increasing *Thioredoxin H3* (*Trxh3*) expression and antioxidant activities (Table 3) [104]. This improvement alleviated plant cellular damage and enhanced aluminum stress resilience. In contrast, Arabidopsis loss-of-function mutation of GSNOR, *gsnor1/hot5* mutant, exhibited resistance to oxidative and ER stress [42]. The ER is a key protein-folding compartment, where one-third of all eukaryotic proteins are translocated from the cytosol to acquire their appropriate conformations. Upon environmental stress, increased protein loading affects ER homeostasis, resulting in unfolded or misfolded proteins. The *gsnor1/hot5* mutant exhibits increased expression of ER-stress-responsive genes (*BiPs*, *ERDJ3A*, and *SHD*), suggesting a pre-activated stress state that renders the mutant insensitive to Tm-induced ER stress (Figure 2; Table 2). ER usually induces ROS production, where Qin et al. [42] identified that endogenous NO in *gsnor1/hot5* reduced oxidative damage by activating S-nitrosylation of ER OXIDOREDUCTASE 1 (ERO1*^Cys337^*), which promotes disulfide bond formation and protein folding (Figure 2; Table 1 and Table 2). Collectively, these findings highlight a crucial function for GSNOR-mediated redox switches in regulating ER function in plants.

**Table 3:** GSNOR-mediated regulation of abiotic and biotic stress tolerance in crops.

Species	Mode of Genetic Modification	Stress Conditions	GSNOR Activity/Expression	NO/SNO Levels	Crosstalk with Other Proteins	Stress Effects	ROS: Antioxidant	Crosstalk with Hormones	Reference
*Solanum lycopersicum* L.	*GSNOR*	Saline–Alkali Stress	–	Decreased/Decreased	HA2	Tolerance	↓:–	Melatonin	[101]
	*GSNOR-RNAi*			Increased/Increased		Sensitive	↑:–		
*Nicotiana tabacum*	*GSNOR1*	Aluminum Stress	Induced	Decreased/Decreased	Trxh3	Tolerance	↓:↑	–	[104]
*Solanum lycopersicum* L.	*GSNOR-RNAi*	Salt Stress	–	–	P5CR	Sensitive	–	–	[54]
*Solanum lycopersicum* cv. Ailsa	*GSNOR-RNAi*	Salt Stress	–	–	ACOh4	Sensitive	–	Ethylene	[56]
*Ganoderma lucidum*	*GSNOR-RNAi*	Heat Stress	Inhibited	–	CAT	Tolerance	↓:↑	–	[105]
*Solanum lycopersicum* L.	*GSNOR-RNAi*	Salt Stress	–	Increased/–	MAPK3, ACO1	Sensitive	↑:–	–	[106]
*Solanum lycopersicum* L. cv. Ailsa	*GSNOR-Silenced*	High-Temperature Stress	Inhibited	–/Increased	RBOH1	Sensitive	↑:↓	ABA and SA	[63]
*Nicotiana tabacum*	*GSNOR*	Nitrate Stress	Induced	Decreased/Decreased	–	Tolerance	↓:↑	–	[92]
*Solanum lycopersicum* L.	*GSNOR*	Fe Deficiency Stress	Induced	Decreased/Decreased	–	Tolerance	↓:↑	–	[78]
*Solanum lycopersicum* L. cv. Condine Red	*GSNOR-Silenced*	Cold Acclimation	Inhibited	Increased/	NR, MPK1/2	Tolerance	–	–	[72]
*Solanum lycopersicum* L.	*GSNOR*	Alkaline Stress	Induced	Decreased/Decreased	–	Tolerance	↓:↑	–	[100]
	*GSNOR-Suppressed*		Inhibited	Increased/Increased		Sensitive	↑:↓		
*Oryza sativa*	*GSNOR*	Oxidative Stress	Induced	–/Decreased	–	Tolerance	↑:↓	–	[107]
	*GSNOR-RNAi*		Inhibited	–/Increased		Sensitive	–		
*Solanum lycopersicum* L.	*GSNOR*	Botrytis cinerea	–	Decreased/Decreased	COMT2	Sensitive	–	JA, Melatonin	[108]
	*GSNOR-RNAi*			Increased/Increased		Tolerance			
	*GSNOR-Silenced*								
*Solanum lycopersicum* L.	*gsnor*	*P. capsici*, flg22	Inhibited	–	PcRD18, ATG8c	Sensitive	↓:–	SA	[109]
	*GSNOR-silenced*								
*Solanum lycopersicum* L.	*GSNOR*	Botrytis cinerea	Induced	Decreased/–	–	Tolerance	–	–	[110]
	*GSNOR-RNAi*		Inhibited	Increased/–		Sensitive			
*Solanum lycopersicum* L.	*GSNOR*	*Pst* DC3000	Induced	–	–	Tolerance	–	–	[111]
*Solanum lycopersicum* L.	*GSNOR*	*Pst* DC3000	Induced	–	PR1	Tolerance	–	SA	[112]
	*GSNOR-RNAi*		Inhibited			Sensitive			
*Medicago truncatula* L.	*35S:GSNOR*	*Aphanomyces euteiches*	Induced	–/Increased	NR	Tolerance	–	–	[113]

Full abbreviations from the table can be found in the main text or Abbreviation section. ↑, increased; ↓, decreased.

## 3. Changes in GSNOR Activity Affect Biotic Stress Tolerance

**Figure 4:**
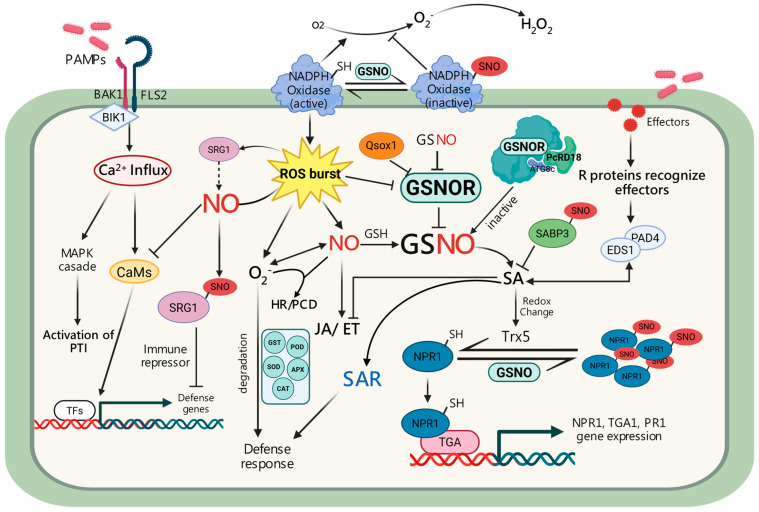
This figure illustrates the integration of NO and GSNOR signaling into plant immunity. Recognition of pathogen-associated molecular patterns (PAMPs) by receptor-like kinases such as FLS2 and BAK1 activates the cytoplasmic kinase BIK1, triggering Ca^2+^ influx, MAPK cascades, and NADPH oxidase (RBOHD)-dependent ROS bursts. Nitric oxide (NO) interacts with ROS to regulate hypersensitive response (HR) and programmed cell death (PCD), and reacts with glutathione (GSH) to form S-nitrosoglutathione (GSNO), a mobile reservoir of NO bioactivity and the main donor of S-nitrosylation (SNO). GSNO levels are tightly controlled by S-nitrosoglutathione reductase (GSNOR), while regulators such as QSOX1 and PcRD18 modulate its activity. Protein S-nitrosylation modifies immune regulators, including SRG1, SABP3, and NPR1, thereby controlling their stability, activity, and redox-dependent transitions. NPR1, in particular, undergoes S-nitrosylation to regulate its oligomer–monomer transition and SA-dependent transcriptional activity (Figure 4; Table 1 and Table 4). Denitrosylation is mediated by thioredoxin h5 (Trx5), linking NO/SNO homeostasis to SA signaling. Pathogen effectors can suppress immunity by targeting GSNOR, whereas R proteins and the EDS1–PAD4 complex activate effector-triggered immunity (ETI). Through the GSNO/GSNOR balance, NO signaling intersects with salicylic acid (SA), jasmonic acid (JA), and ethylene (ET) pathways, coordinating defense gene expression, HR/PCD, and systemic acquired resistance (SAR). Black solid arrows indicate direct activation where blunt shows inhibition.

A defining feature of plant immunity is the dynamic reprogramming of cellular redox homeostasis, shaped by the interplay between ROS and RNS. Within this mechanism, NO has emerged as a central signaling molecule that orchestrates defense responses against diverse biotic stresses. As previously discussed, the NO moiety transferred to a reactive cysteine residue is central to a redox-based PTM, S-nitrosylation, which regulates protein function and immune defense. This entire process is controlled by GSNOR, which acts as a critical checkpoint in NO signaling for plant immunity (Figure 4; Table 3 and Table 4).

S-nitrosylation modifies a suite of immune-related proteins, including RBOHD, NPR1, TGA1, SABP3, and peroxiredoxins, thereby wiring NO signaling into both pattern-triggered immunity (PTI) and effector-triggered immunity (ETI) [50,51,52,114,115]. Importantly, this modification can act as a double-edged sword. While host cells employ it to fine-tune defense, pathogens may exploit S-nitrosylation machinery to suppress immunity and promote virulence. NO mediates PTI through stage-specific S-nitrosylation of immune regulators. Recognition of pathogen-associated molecular patterns (PAMPs) by receptor-like kinases such as FLS2 activates BIK1, which phosphorylates the NADPH oxidase RBOHD to induce ROS bursts (Figure 4; Table 4) [39]. Early S-nitrosylation of BIK1*^Cys80^* enhances its stability and phosphorylation, thereby amplifying ROS production, whereas later modification of RBOHD*^Cys890^* suppresses ROS output to prevent excessive oxidative stress (Figure 4; Table 1 and Table 4) [30]. This temporal regulation demonstrates how NO balances immune activation with cellular homeostasis. In *atgsnor1* mutants, excessive SNO accumulation disrupts SA-dependent transcriptional reprogramming, impairs NPR1 monomerization, and compromises basal and nonhost resistance (Figure 4; Table 4). Conversely, GSNOR overexpression restores redox balance and enhances immune responses. These results underscore GSNOR as a critical metabolic checkpoint.

Additionally, HMAD1 negatively regulates immunity by modulating GSNOR activity [116], whereas QSOX1, a redox-sensitive enzyme, interacts with both GSNOR and RBOHD to adjust ROS/RNS balance, limiting excessive cell death while sustaining basal defense (Figure 4; Table 4) [65]. At the nuclear level, SRG1, a zinc-finger transcription factor, is activated by pathogen-induced NO bursts to repress negative regulators of defense [117]. Subsequent S-nitrosylation of conserved cysteines (e.g., Cys87) disrupts Zn^2+^ coordination and DNA binding, releasing SRG1 repression in a redox-controlled feedback loop. GSNOR-mediated SNO homeostasis is pivotal in fine-tuning this regulatory switch (Figure 4; Table 1 and Table 4). NO also modulates other immune regulators in *Arabidopsis*. S-nitrosylation of NPR1*^Cys156^* promotes oligomerization and protein stability, whereas modification of SA-binding protein 3 (AtSABP3*^Cys280^*) reduces SA binding and carbonic anhydrase activity, thereby providing negative feedback to SA-mediated immunity (Figure 4; Table 1 and Table 4) [51]. Moreover, NO/GSNOR signaling intersects with abiotic and biotic stress pathways. The aldehyde oxidase gene *AtAO3*, involved in ABA biosynthesis, antagonizes SA signaling while promoting drought tolerance through ABA-dependent stomatal closure [118]. Accordingly, *atao3* mutants show elevated *PR1* expression and hypersensitive response (HR), illustrating the antagonistic interplay between ABA and SA, with NO functioning as a molecular integrator of biotic and abiotic stress signaling.

Pathogens exploit this hub to suppress host immunity. For example, Phytophthora effectors directly inhibit GSNOR, disrupting SA-dependent transcriptional activation and ROS bursts (Figure 4; Table 4) [119]. The RxLR effector PcRD18 further promotes autophagic degradation of GSNOR via ATG8c, elevating SNO accumulation, suppressing ROS production, and ultimately enhancing pathogen virulence. These regulatory mechanisms are conserved in crops. In wheat, pathogen-induced expression of *TaNIA* and *TaGSNOR* correlates with increased NO production and dynamic GSNO turnover, suggesting roles in both PTI and ETI [120]. In tomato, *SlGSNOR* overexpression enhances resistance to *Pseudomonas syringae* pv. *tomato* and promotes fruit development [112], while silencing *SlGSNOR* compromises SA-dependent defense [111]. In rice, the *noe1* mutant, defective in catalase, shows uncontrolled NO/ROS accumulation, whereas *OsGSNOR* overexpression restores redox balance and prevents runaway cell death [107]. Even fungal pathogens such as *Magnaporthe oryzae* rely on GSNOR-mediated denitrosylation for infection, as loss of GSNOR impairs appressorium formation, turgor generation, and virulence [121]. Collectively, these studies highlight the role of GSNOR as a conserved regulatory hub across plant species. By controlling NO bioactivity and S-nitrosylation, GSNOR can coordinate immune responses, prevent nitrosative stress, and balance defense with plant growth.

**Table 4:** GSNOR-mediated regulation of biotic stress tolerance in *A. thaliana*.

Mode of Genetic Modification	Stress Conditions	GSNOR Activity/Expression	NO/SNO Levels	Crosstalk with Other Proteins	Stress Effects	ROS: Antioxidant	Crosstalk with Hormones	Reference
*gsnor1-3*	*Pst* DC3000 hrcC^−^, flg22	–	–/Increased	BIK1, RBOHD, FLS2, BAK1	Sensitive	↑:–	–	[39]
*par2-1*								
*gsnor1-1*	Phytophthora parasitica	–	–	–	–	–	–	[119]
*gsnor1-3*					Sensitive	↓:–	SA	
*par2-1*						–	–	
*gsnor1-3*	*Pst* DC3000 (avrRpt2), *Pst* DC3000 (avrRpm1), *Pst* DC3000 (avrRps4)	Inhibited	–/Increased	RBOHD	Sensitive	–	–	[65]
*gsnor1-3*	*Pst* DC3000 (avrB)	–	–/Increased	–	Sensitive	–	SA	[118]
*gsnor1-3*	*Pst* DC3000, *Pst* DC3000 (avrRpm1)	–	–/Increased	SRG1	Sensitive	↓:–	SA	[117]
gsnor1-3	*Pst* DC3000, *Pst* DC 3000 (avrB)	–	–/Increased	–	Sensitive	–	SA	[116]
*gsnor1-1*	*Pst* DC3000 (avrB), *Pst* DC3000 (avrRps4), *Pst* DC3000 (virulent), Psp	–	Decreased/Decreased	–	Tolerance	–	–	[122]
*35S:FLAG-GSNOR1*			–					
*gnsor1-3*		Inhibited	Increased/Increased		Sensitive		SA	
*par2-1*		–	–				–	
*gsnor1-1*	*Pst* DC3000 (avrB), *Pst* DC3000 (avrRps4), *Pst* DC3000, *H. arabidopsidis* Emwa1	Induced	Decreased/Decreased	RBOHD	Sensitive	↑:–	SA	[50]
*gnsor1-3*		Inhibited	Increased/Increased		Tolerance	↓:–		
*gsnor*	*Pst* DC3000 (avrRpt2), *Pst* DC3000	Inhibited	–	–	–	–:↑	–	[123]
*gsnor1-1*	*Pst* DC3000 (avrB)	–	–/Decreased	SABP3	Tolerance	–	SA	[51]
*gsnor1-3*			–/Increased		Sensitive			
*gsnor1-1*	*Pst* DC3000 (avrB), *Pst* DC3000	Induced	–/Decreased	–	Tolerance	–	SA	[30]
*gsnor1-2*								
*gsnor1-3*		Inhibited	–/Increased		Sensitive			

Full abbreviations from the table can be found in the main text or Abbreviation section. ↑, increased; ↓, decreased.

## 4. Nitric Oxide for Innovative Agricultural Application: Potential Nanotechnology and Its Limitations

**Figure 5:**
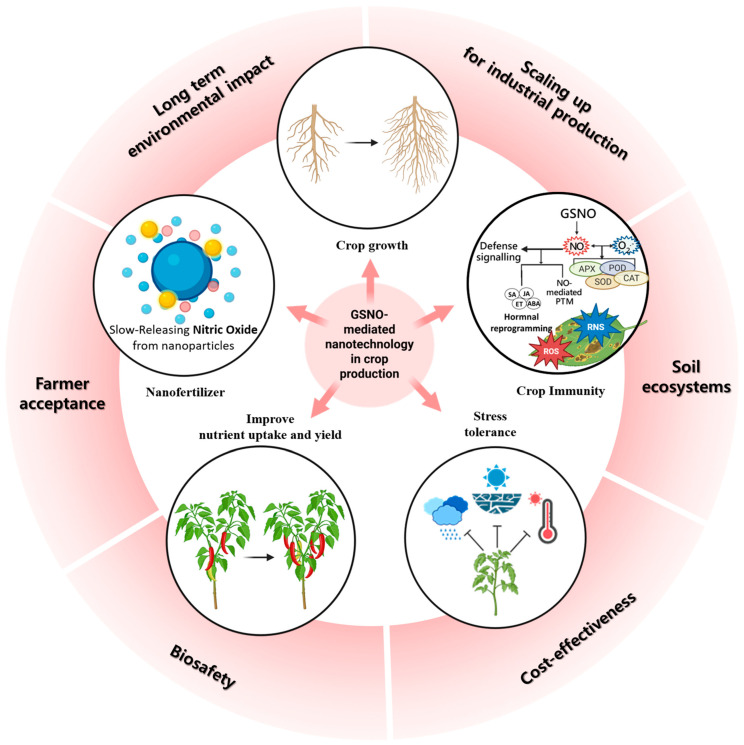
The figure illustrates the potential of nitric-oxide-based nanotechnology in agriculture, including (i) controlled and slow release of nitric oxide (NO) from nanoparticles, (ii) enhancement of plant defense signaling and stress tolerance through modulation of reactive oxygen/nitrogen species (ROS/RNS) and hormone crosstalk, and (iii) improvements in growth and productivity under abiotic and biotic stresses. Moreover, this figure highlights key limitations and challenges that must be addressed for field application: (i) scaling up nanoparticle production for industrial use, (ii) understanding impacts on soil ecosystems and microbial interactions, (iii) ensuring cost-effectiveness for farmers, (iv) biosafety and risk assessment, (v) farmer acceptance and regulatory approval, and (vi) evaluation of long-term environmental impacts.

Recent advances in nanotechnology have expanded the potential of smart fertilizers by providing novel platforms for precise nutrient delivery [124,125]. In the previous sections, we discussed the various roles of NO in plant physiology and its involvement in developmental processes. Despite its considerable applications, its utility is limited by a short shelf life due to its rapid redox nature in the presence of light, high temperatures, and catalytic metal ions [126,127,128]. Therefore, by believing in its importance, studies focusing on the creation of innovative NO-based nanomaterials could be applied to basic and applied plant research (Figure 5) [129]. This review, while focused on GSNOR’s role in NO signaling, also explores NO-based nanoparticles (NPs), especially those using GSNO as a donor. Ultimately, we examine the significance of GSNOR in the context of nano-based agricultural solutions.

NO-based NPs have been shown to improve NO’s shelf-life and provide a slow release mechanism (Figure 5) [130,131,132,133]. To date, most NO-based NPs have been encapsulated with chitosan-based NPs, which increases NO stability [134]. NO release from these encapsulated nanocarriers improves plant resilience to salt and drought stress. These tolerance mechanisms are involved in increasing plant biomass, photosynthesis, ROS detoxification, antioxidant properties, and crop yield compared to NO donors alone (Figure 5) [131,132,135,136]. Together with these findings, a recent study showed that encapsulation of GSNO into chitosan nanocapsules applied in *Brassica napus* promoted stable SNO accumulation and redox balance while minimizing nitrosative stress [133]. Intriguingly, this NP exhibited NO release for more than 4 h, demonstrating the potential of nano-formulated NO availability. In line with the NO release, GSNO–chitosan NPs inactivated GSNOR activity in the seedling of *B. napus*, where Methela et al. [132] reported that the relative expression of *GSNOR1* in soybean shoots was increased upon drought stress. These results suggest that slow-release GSNO–chitosan NPs do not trigger GSNOR activity, while drought stress forces GSNOR to break down the excess NO to balance ROS and RNS for plant tolerance under stress. Hence, we speculate that such improvised NO-based nanocarriers are building a strong field in NO-based nanotechnology for future agricultural applications.

Despite the promising potential of nanotechnology-based smart fertilizers and NO-delivery platforms such as HAs and chitosan NPs, several limitations remain before their widespread application in agriculture can be realized (Figure 5). First, the large-scale synthesis, stability, and cost-effectiveness of NP-based formulations are still major challenges, as production methods often require complex processes that may not be easily scalable for field use. Second, the long-term environmental and ecological impacts of nanomaterials in soil ecosystems are not fully understood [137,138]. Potential concerns include the persistence and accumulation of NPs, as well as their unintended interactions with soil microbiota [139,140]. These interactions may affect crucial soil processes such as nutrient cycling, the structure of microbial communities, and beneficial functions like nitrogen fixation or phosphate solubilization [139,140]. For example, studies have shown that low concentrations of silver NPs can adversely affect plant species and significantly alter bacterial community composition and enzyme activities in soil [141].

Nanomaterials can enter soil and aquatic ecosystems through various routes, including their presence in industrial products, environmental cleanup technologies, and unintentional releases from air, water, and sewage sludge applications (Figure 5) [142,143]. Engineered nanomaterials (ENMs) are expected to accumulate significantly in terrestrial environments, with biosolids from wastewater treatment serving as a major pathway for their introduction into the environment, exposing important soil microorganisms [144]. The unique properties of NPs, such as high surface area and reactivity, can lead to environmental hazards and potentially harm soil health (Figure 5) [143,145]. Another limitation lies in the complexity of NO/GSNOR signaling itself. While controlled NO release provides multiple physiological benefits, excessive or misregulated NO levels can lead to nitrosative stress, disrupting redox balance and impairing plant growth [146]. Therefore, fine-tuning NO dosage, release kinetics, and interactions with GSNOR pathways remains a critical challenge for practical implementation. In addition, crop-specific responses to NO and GSNO delivery need to be carefully evaluated, as species-dependent differences may influence the efficiency and outcomes of such interventions [147]. Finally, regulatory frameworks, biosafety assessments, and farmer acceptance of nanotechnology-based fertilizers are still in the early stages of development [138,148]. To achieve successful implementation in the field, interdisciplinary efforts are required, combining expertise from plant physiology, soil microbiology, materials science, systems biology, and agricultural policy [138,149]. Addressing these multifaceted challenges is crucial to fully harness the potential of smart NO-delivery systems for sustainable and climate-smart agriculture, and will likely improve our understanding of the GSNOR response [148,150].

## 5. Conclusions and Future Perspectives

Understanding the role of GSNOR in plant biology is crucial for stress tolerance under changing climatic conditions. A wide range of studies on NO signaling have been carried out with the endogenous modification of GSNOR activity in model plants and crops. This review aims to consolidate current knowledge on GSNOR’s involvement in plant tolerance to both abiotic and biotic stresses while highlighting key knowledge gaps. While stress tolerance depends on multiple variables, GSNOR shows pleiotropic effects on it. It is established that GSNOR controls global S-nitrosylation, which primarily contributes to resilience against abiotic stress but shows negligible efficacy against plant pathogenicity. However, the precise mechanism by which GSNOR confers stress tolerance needs to be revisited. For instance, (i) why cellular GSNO inhibits heat tolerance but S-nitrosylated proteins exhibit enhanced tolerance needs to be explored; (ii) the regulatory negative effect between NO/GSNOR and ABA under drought conditions is insufficiently studied; (iii) when elevated GSNO is present in the loss-of-function mutation of GSNOR, how it modulates plant nutrient availability and uptake is poorly investigated; (iv) except for seed germination status in the loss-of-function mutation of GSNOR under low-O_2_ conditions, its survivability and signaling are completely overlooked; (v) despite the knowledge of auxin-dependent growth and development of GSNOR-mutated plants, their crosstalk in response to stress tolerance is still enigmatic, and (vi) there is insufficient knowledge on NO/GSNOR-mediated PAMP recognition for plant immunity. Addressing these critical gaps will be crucial for improving GSNOR-dependent crop growth, stress resilience, and productivity, particularly under future climate challenges, and may also be informative in the development of NO-based agricultural technologies.

## Data Availability

No new data were created or analyzed in this study. Data sharing is not applicable to this article.

## Supplementary Materials

The following supporting information can be downloaded at: https://www.mdpi.com/article/10.3390/ijms262311486/s1.

## Abbreviations

The following abbreviations are used in this manuscript:

ADH3	Class III alcohol dehydrogenase
APX1	Ascorbate Peroxidase 1
BIK1	Botrytis-induced kinase 1
COP1	Constitutive Photomorphogenesis Protein 1
Cys	Cystein
ER	Endoplasmic Reticulum
ERO1	Endoplasmic Reticulum Oxidoreductin 1
ET	Ethylene
ETI	Effector-Triggered Immunity
FRO1	Ferric-Reductase Oxidase1
GABA-TP1/2	Gamma-aminobutyrate Transaminase 1/2
GSH	Reduced Glutathione
GSNO	S-nitrosoglutathione
GSNOR	S-nitrosoglutathione Reductase
GSSG	Glutathione disulfide
HDA19	Histone Deacetylase 19
HFR1	Far-red elongated hypocotyl 1
JA	Jasmonic Acid
MDHAR	Monodehydroascorbate Reductase
NADH	Nicotinamide adenine dinucleotide
NIA1/2	Nitrate Reductase 1/2
NO	Nitric Oxide
NOA1	Nitric Oxide-Associated 1
NOS	Nitric Oxide Synthase
NOX1	NADPH oxidase 1
NPR1	Nonexpressor of Pathogenesis-Related genes 1
NRAMP3/4	Natural Resistance-Associated Macrophage Protein 3/4
OPT	Oligopeptide transporter
P5CR	Pyrroline-5-Carboxylate Reductase
PDC1	Pyruvate Decarboxylase 1
PIC1	Permease in Chloroplasts 1
QSOX1	Quiescin Sulfhydryl Oxidase 1
RBOHD	Respiratory Burst Oxidase Homologue D
RGA	Repressor of Gibberellin 1-3
RNS	Reactive Nitrogen Species
ROS	Reactive Oxygen Species
SA	Salicylic Acid
SABP3	Salicylic Acid-Binding Protein 3
SAR	Systemic Acquired Resistance
SNO	S-nitrosothiol
SNP	Single-Nucleotide Polymorphism
SnRK	Sucrose Non-Fermenting 1-related Protein Kinase 2.6
Trxh3	Thioredoxin H3
