# Supplementary material for: The Central Role of GSNOR: Decoding Nitric Oxide Signaling for Crop Stress Tolerance
Source: Int J Mol Sci. 2025 Nov 27;26(23):11486. doi: 10.3390/ijms262311486 (PMC12692127; doi:10.3390/ijms262311486)
Supplement: Supplementary file 1 [file ijms-26-11486-s001.zip › ijms-3943143-supplementary.pdf]

The Central Role of GSNOR: Decoding Nitric Oxide Signaling for Crop Stress Tolerance

Ashim Kumar Das [1,†], Da-Sol Lee [1,†], Geum-Jin Lee [1], Ye-Song Kim [1], Sajeel Hussain [1], Moon-Sub Lee [2], Byung-Wook Yun [1] and Bong-Gyu Mun [3,*]

[1] Department of Applied Biosciences, College of Agriculture and Life Sciences, Kyungpook National University, Daegu 41566, Republic of Korea
[2] Department of Crop Science, Chungbuk National University, Cheongju 28644, Republic of Korea
[3] Department of Environmental and Biological Chemistry, Chungbuk National University, Cheongju 28644, Republic of Korea
* Correspondence: munbg@chungbuk.ac.kr

Table S1. List of species and accession number used in the phylogenetic tree.

Accession Number	Gene Name	Species Name
NP_001238796.1	SlGSNOR	*Solanum lycopersicum*
XP_015088229.1	SpGSNOR	*Solanum pennellii*
NP_001274960.1	StGSNOR	*Solanum tuberosum*
XP_016541792.1	CaGSNOR	*Capsicum annuum*
KAK4418454.1	SaGSNOR	*Sesamum alatum*
XP_027160260.1	CeGSNOR	*Coffea eugenioides*
XP_021629320.1	MeGSNOR	*Manihot esculenta*
XP_047981491.1	ShGSNOR	*Salvia hispanica*
XP_002301836.4	PtGSNOR	*Populus trichocarpa*
XP_042014657.1	SsGSNOR	*Salvia splendens*
KAK8982377.1	HsGSNOR	*Hibiscus sabdariffa*
XP_061969073.1	PnGSNOR	*Populus nigra*
NP_199207.1	AtGSNOR	*Arabidopsis thaliana*
XP_010494387.1	CsGSNOR	*Camelina sativa*
AQM74409.1	BjGSNOR	*Brassica juncea*
XP_006280634.1	CrGSNOR	*Capsella rubella*
XP_009101752.1	BrGSNOR	*Brassica rapa*
XP_013733432.1	BnGSNOR	*Brassica napus*
XP_006403162.1	EsGSNOR	*Eutrema salsugineum*
XP_018485206.1	RsGSNOR	*Raphanus sativus*
CAN6874728.1	BoGSNOR	*Brassica oleracea*
KAF8104459.1	SaGSNOR	*Sinapis alba*
XP_003537123.1	GmGSNOR	*Glycine max*
BAF63879.1	OsGSNOR	*Oryza sativa Japonica Group*
ACG34695.1	ZmGSNOR	*Zea mays*
PRW60671.1	CsorGSNOR	*Chlorella sorokiniana*
PSC68961.1	McGSNOR	*Micractinium conductrix*
PXF48590.1	GcADH3	*Gracilariopsis chorda*
KAA8492794.1	PpADH3	*Porphyridium purpureum*
GJD07100.1	GsADH3	*Galdieria sulphuraria*
AAP51052.1	SpADH3	*Saccharomyces pastorianus*
AAP51044.1	SbADH3	*Saccharomyces bayanus*
KAK4332799.1	RtADH3	*Rhodotorula toruloides*

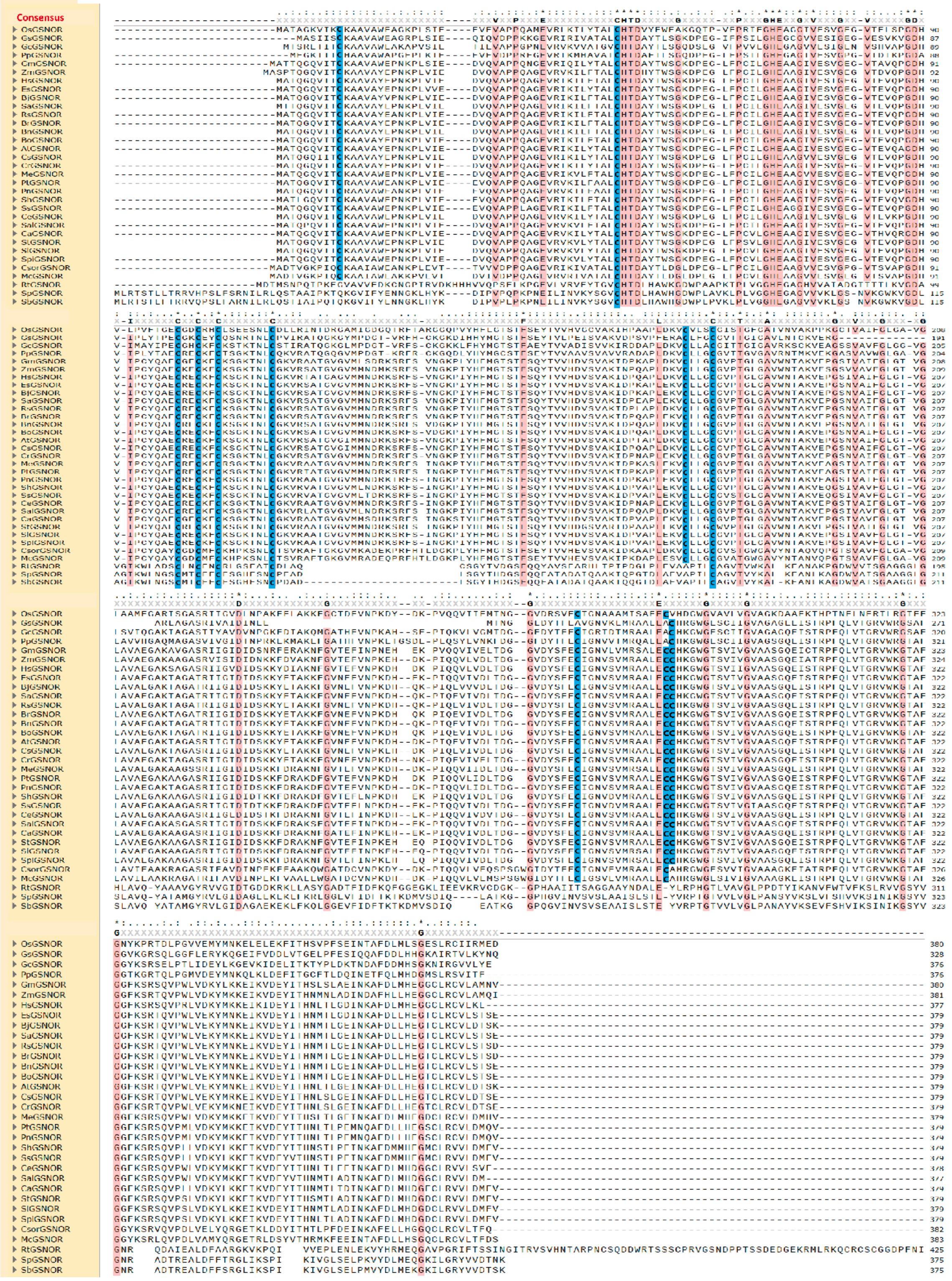

Figure S1. Multiple sequence alignment of selected species. The protein sequences of GSNOR gene in selected species were downloaded from NCBI (https://www.ncbi.nlm.nih.gov/) using pblast and were aligned on EMBL-EBI (https://www.ebi.ac.uk/jdispatcher/msa) using Clustal W. Downloaded multiple sequence alignment was viewed on SnapGene 8.1.0 at >95% sequence conservation level.
